# Mitochondrial Swelling and Incipient Outer Membrane Rupture in Preapoptotic and Apoptotic Cells

**DOI:** 10.1002/ar.22553

**Published:** 2012-08-21

**Authors:** A Sesso, JE Belizário, MM Marques, ML Higuchi, RI Schumacher, A Colquhoun, E Ito, J Kawakami

**Affiliations:** 1 Setor de Biologia Estrutural, Laboratório de Imunopatologia, Instituto de Medicina Tropical, Universidade de São PauloSão Paulo, Brazil; 2 Departamento de Farmacologia, Instituto de Ciências Biomédicas, Universidade de São PauloSão Paulo, Brazil; 3 Departmento de Dentística, Faculdade de Odontologia, Universidade de São PauloSão Paulo, Brazil; 4 Laboratório de Anatomia Patológica, Instituto do Coração, Universidade de São PauloSão Paulo, Brazil; 5 Departamento de Bioquímica, Instituto de Química, Universidade de São PauloSão Paulo, Brazil; 6 Departamento de Histologia, Instituto de Ciências Biomédicas, Universidade de São PauloSão Paulo, Brazil

**Keywords:** electron microscopy, mitochondria, apoptosis, rupture of the outer mitochondrial membrane, focal matrix herniation in mitochondria, inner mitochondrial membrane, mitochondrial outer membrane permeabilization, mitochondrial proteins of the intermembrane space, mitochon-drial permeability transition, outer mitochon-drial membrane

## Abstract

Outer mitochondrial membrane (OMM) rupture was first noted in isolated mitochondria in which the inner mitochondrial membrane (IMM) had lost its selective permeability. This phenomenon referred to as mitochondrial permeability transition (MPT) refers to a permeabilized inner membrane that originates a large swelling in the mitochondrial matrix, which distends the outer membrane until it ruptures. Here, we have expanded previous electron microscopic observations that in apoptotic cells, OMM rupture is not caused by a membrane stretching promoted by a markedly swollen matrix. It is shown that the widths of the ruptured regions of the OMM vary from 6 to 250 nm. Independent of the perforation size, herniation of the mitochondrial matrix appeared to have resulted in pushing the IMM through the perforation. A large, long focal herniation of the mitochondrial matrix, covered with the IMM, was associated with a rupture of the OMM that was as small as 6 nm. Contextually, the collapse of the selective permeability of the IMM may precede or follow the release of the mitochondrial proteins of the intermembrane space into the cytoplasm. When the MPT is a late event, exit of the intermembrane space proteins to the cytoplasm is unimpeded and occurs through channels that transverse the outer membrane, because so far, the inner membrane is impermeable. No channel within the outer membrane can expose to the cytoplasm a permeable inner membrane, because it would serve as a conduit for local herniation of the mitochondrial matrix. Anat Rec, 2012. © 2012 Wiley Periodicals, Inc.

The central event of the mitochondrial-dependent apoptotic pathway is the release of mitochondrial proteins of the intermembrane space (MPIS) into the cytoplasm. These proteins initiate and execute the actual process of cell demolition. They are released from the mitochondria by one of two possible ways. One of the exit mechanisms is through the rupture of the outer mitochondrial membrane (OMM) (Petit et al., 1996, 1998; Skulachev, 1996; Scarlett and Murphy, 1997). The second exit model proposes the formation or opening, or both, of special channels in an unruptured OMM, a process termed mitochondrial membrane permeabilization (Brenner and Kroemer, 2000; Kroemer et al., 2007), or mitochondrial outer membrane permeabilization (MOMP) (Chipuk et al., 2006; Green, 2006).

According to the first paradigm, rupture of the OMM is preceded by the mitochondrial permeability transition (MPT), a sudden loss in the selective permeability of the inner mitochondrial membrane (IMM). In the majority of cells entering in apoptosis this permeabilization of the IMM is an early apoptotic event promoted by the sustained aperture of the MPT pore, a proteinaceous channel or pore situated between both mitochondrial membranes. The size of the diameter of the pore makes it possible for cytoplasmic fluids to traverse the IMM, thereby swelling the matrix. The first proposal (Skulachev, 1996) to explain how the OMM ruptures was based on observations of isolated mitochondria (Petit et al., 1996) in the state of permeability transition. The MPT-induced swelling of the matrix progressively engorges this compartment, spreading out its IMM lining, which uses the membrane stored in the cristae. The OMM, having a smaller surface area than the IMM, ruptures when its distension limit is exceeded. By inference, it has long been assumed that this mechanism also occurs *in situ*. The transitory swelling observed in normal mitochondria of living cells is caused by a short-lived and partial MPT. If the MPT pore is irreversibly kept open, cell death will occur (reviewed in Zoratti et al., 2005; Rasola and Bernardi, 2007).

In mitochondria with a ruptured outer membrane, both the mitochondrial matrix and the IMM protrude into the cytoplasm through the perforation, producing a focal hernia on the surface of the organelle (Angermüller et al., 1998, Kwong et al., 1999, Feldman et al., 2000, Sesso et al., 2004; Sesso, 2006; Vogler et al., 2008). Thus, rupture of the OMM promotes the release of cytochrome *c* into the cytoplasm (Feldman et al., 2000; Vogler et al., 2008).

In the present study, we extend our observations of minute breaches of the OMM in mitochondria from apoptotic cells whose matrices often exhibit a small or a moderate degree of swelling (Sesso, 2006). The conclusions of these studies differ considerably from those in previous models (Skulachev, 1996; Green and Reed, 1998; Desagher and Martinou, 2000; Martinou and Green, 2001; Reed and Green, 2002; Ly et al., 2003; Machida and Osada, 2003; Robertson et al., 2003).

## MATERIALS AND METHODS

Cell types and respective treatments, fixation, embedding procedures, and ultra-thin sectioning have been published previously (Sesso et al., 1999, 2004). We have re-examined many electron micrographs and, under the microscope, previously sectioned cells. In some cases, earlier embedded cells were re-sectioned for further examination.

### Cell Lines and Apoptogenic Agents

(1) PC-12 cells (pheochromocytoma, rat, ATCC CRL-172) purchased from the American Type Culture Collection (ATCC) were cultured in RPMI-1640 85% with 5% fetal bovine serum and 10% heat-inactivated horse serum (56°C for 30 min) and 1% antibiotic-antimycotic solution in flasks coated with poly-l-lysine (50 μg/mL) (Sigma). The flasks were incubated at 36°C in a humidified atmosphere in 7.5% CO_2_. Apoptosis was induced by sera deprivation for 2, 4, 6, 8, 16, 24, and 48 hr. We obtained 26 successive sections from a cell pellet prepared from cells deprived of sera for 16 hr. Serial sections were prepared from cells deprived of sera for 4 or 16 hr. Nonconsecutive serial sections were collected as follows: every third section was taken after the first and apoptotic cells were examined. (2) A second PC-12 cell line that was developed at the Instituto Butantan in São Paulo (Ho and Raw, 1992) was used. These cells were grown and stabilized in Dulbecco's modified Eagle's medium (DMEM) (Life Technologies, Carlsbad, CA) supplemented with 10% FCS (Cultilab, Brazil) at 37°C in a humidified atmosphere in 5% CO_2._ In contrast to the ATCC PC-12 cell line, these cells attach more readily to the flask surface and do not require a coating of collagen or poly-l-lysine. We refer to this second line as PC-12*. These cells were exposed to 2 μM brefeldin A (BFA) (Calbiochem) and 0.5 μM staurosporine (STS) (Calbiochem) for 16 hr. BFA blocks vesicular transport from the endoplasmic reticulum to the Golgi cisternae but not the retrograde vesicular flux of this circuit. STS is a potent inhibitor of PKC and many other kinases. (3) HL-60 cells (human, peripheral blood, promyelocytic leukemia) (ATCC-CCL-240) exposed to human TNFα (Sigma) 100 ng/mL and camptothecin (Sigma) 2 μM for 6 and 16 hr; these cells were also exposed to actinomycin D (Sigma) 0.25 μg/mL plus cycloheximide 6,25 μg/mL during 16 hr. TNFα is a pro inflammatory cytocine capable of inducing apoptosis. Camptothecin is a DNA topoisomerase that induces apoptosis in cells that are in the S phase of the proliferative cycle. The antibiotic actinomycin D blocks DNA replication and transcription. Cycloheximide inhibits protein synthesis at the level of the ribosomes. (4) BHK-21 (C-13) cells (baby hamster, kidney, ATCC CCL-10) cells were exposed to BFA and STS, and also to human TNFα 40 ng/mL plus cycloheximide (Sigma) 2 μM/16 hr, and to camptothecin 6 μM for 6 or 16 hr. 5-HeLa (epithelioid carcinoma, cervix, human, ATCC CCL-2) cells were exposed to TNFα 10 ng/mL/23 hr.

HL 60 and BHK-21 cells were cultured in RPMI 1640 based medium as for the PC-12* cells with 10% BSA. HeLa cells were grown in DMEM. In all cases the drugs were added to a medium devoid of serum

### Electron Microscopy

Cell types and respective treatments, fixation, embedding procedures, and ultra-thin sectioning have been published previously (Sesso et al., 1999, 2004). In brief, cell pellets not exceeding 1/20–1/40 of the volume of the fixing and washing solutions were fixed with 2% glutaraldehyde (Ladd Research Industries, VT) in 0.1 M PBS at pH 7.4 for 2 hr at 4°C. After a rapid rinse in PBS, cells were fixed again for 1.5 hr in reduced osmium solution prepared by mixing one volume of 2% aqueous osmium tetroxide and one volume of 3% potassium ferrocyanide at room temperature, and contrasted overnight with 0.5% aqueous uranyl acetate. Dehydrated samples were infiltrated by propylene oxide followed by a mixture (1:1, vol/vol) of propylene oxide and an Epon-like mixture, in which Epon was substituted by LX 112 resin (Ladd Research Industries). Samples were embedded in a mixture of NADC methyl anhydride, 16.4 g; dodecenyl succinic anhydride 7.6 g; LX 112 27.1 g; and 2,3,6-tri(dimethylaminomethyl)phenol, 0.75 g (all from Ladd Research Industries). Silver sections contrasted with uranyl acetate and lead citrate (both from Ladd Research Industries) were observed through a binocular eyepiece (10× enlargement) at microscopic magnifications of 5,000 and in the range, 20,000–50,000 with a Jeol 1010 or 1020, Philips 301, or Tecnai 10 electron microscope at 80 kV. In each of the samples, 15–50 apoptotic and at least 100 cells with unaltered or uncharacteristic nuclear structure (CUNS) were examined for mitochondria with ruptured outer membranes.

## RESULTS

**Fig. 1:**
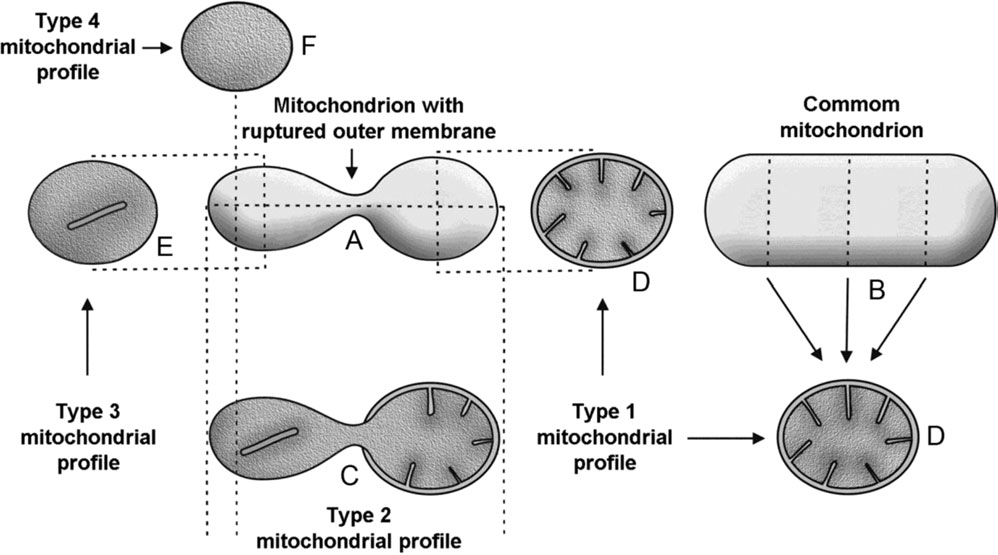
This scheme reproduces Fig. 1 in Sesso et al., 2004 with permission of Wiley-Blackwell. It represents the four types of profiles derived from random sectioning of a mitochondrion with ruptured outer membrane. (**A**) Tri-dimensional representation of a mitochondrion with ruptured outer mitochondrial membrane. (**B**) Tri-dimensional representation of a normal mitochondrion. The schematized profile type 2 is one of the various frontal views of the mitochondrion with ruptured outer membrane (**C**) (inset in Fig. 2). Profile types 3 and 4 are cross sections of the mitochondrial matrix herniated to the cytoplasm and its IMM lining. When these single membrane vesicles contain cristae remnants, they are designated profiles type 3 (**E**). When they do not contain cristae, they are designated profiles type 4 (**F**). Profile type 1 (**D**) may be derived from a mitochondrion with either an intact outer membrane (**B**) or a ruptured one (**C**).

**Fig. 2:**
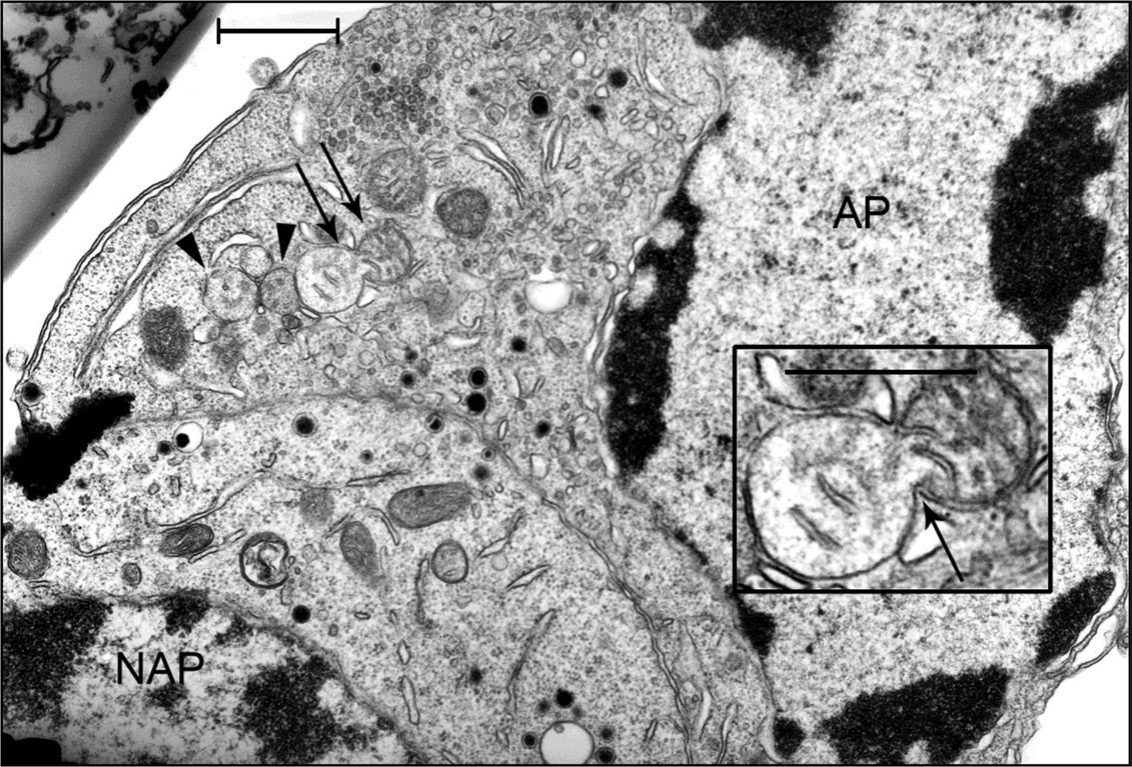
Early structural stage of apoptosis. Nonapoptotic (lower left, NAP) and apoptotic (upper right, AP) PC-12 cells deprived of nutrients for 8 hr. While the chromatin is denser in cell AP than in cell NAP, their cytoplasm has many similar features. However, on closer inspection the polysomes of the apoptotic cells are closer to each other than in the adjoining cell. These changes derive from one of the earliest structural signs of apoptosis, that is decrease in cell size due to water loss caused by increased K^+^ and Cl^−^ ionic effluxes (Shimizu et al., 2004). The inset shows an enlarged view of a mitochondrion with ruptured outer membrane. Through the hole in the OMM, a swollen matrix lined by the IMM herniates (arrow) into the cytoplasm. Arrow heads point to tightly adherent mitochondrial profiles type 3.

**Fig. 3:**
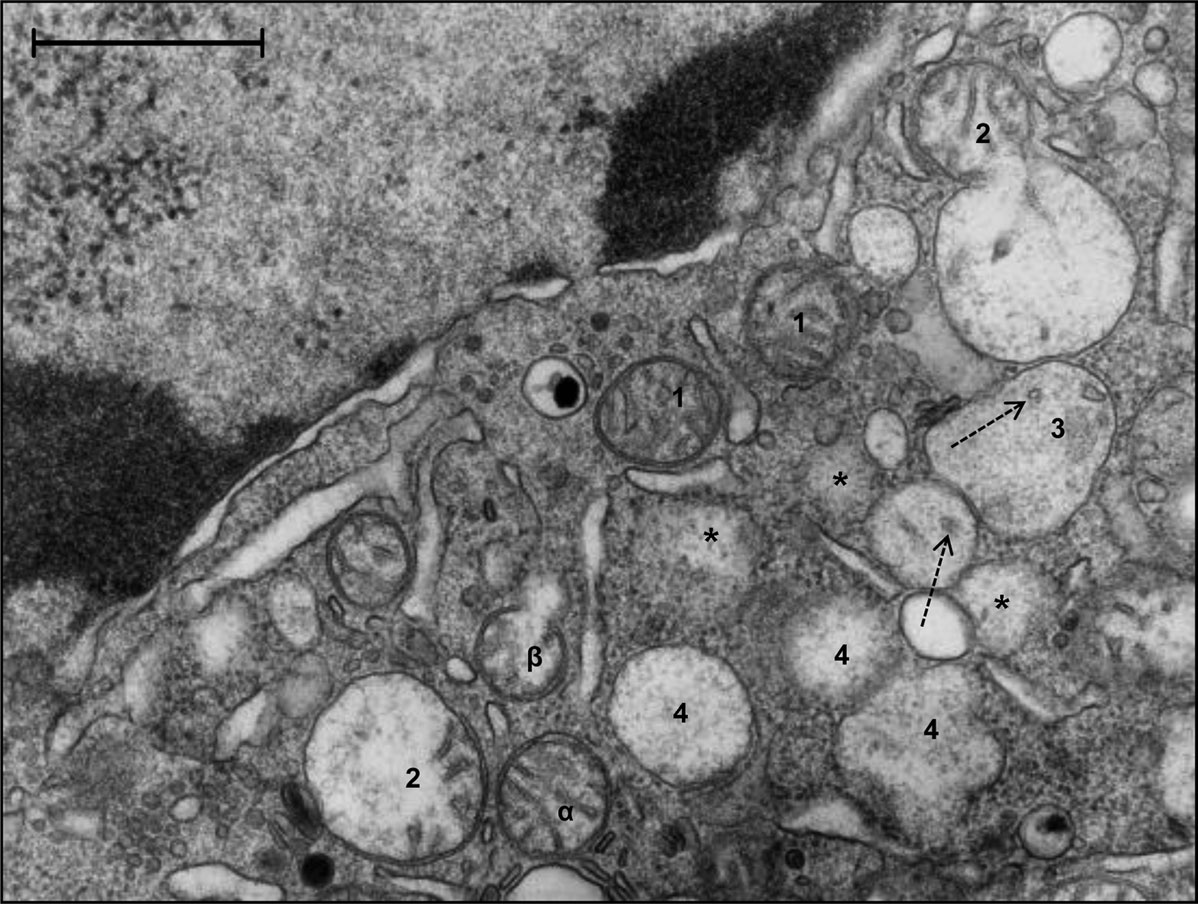
PC-12 cell 4 hr deprived of sera. Profiles showing possible successive stages in the progression of the focal herniation of the mitochondrial matrix lined by the IMM through to the ruptured OMM. Near the typical dense apoptotic chromatin mitochondrial profiles types 1–4 are seen. Profiles α and β seem to represent successive stages of a process of rupture of the OMM. They appear enlarged in panels A and C of Fig. 6. Dashed arrows indicate cross-sections of tubular cristae. Asterisks (*) quite possibly indicate sectors from profiles 2–4 that are partly contained in the thickness of the section.

Random thin sectioning of a mitochondrion with a ruptured outer membrane yields four types of profiles; a general scheme of these mitochondrial profiles, arbitrarily designated profile type 1–4, is presented in Fig. 1. Profile type 1 is indistinguishable from a section of a nearby mitochondrion with an unruptured OMM. The type 2 profile is a frontal view of a mitochondrion with a ruptured outer membrane, exhibiting the region where the OMM was perforated. In this profile type, a swollen matrix, covered by the IMM, is consistently found protruding into the cytoplasm through the perforation caused by the rupture of the OMM. Cross-sections through the focal hernia display single-membrane profiles. Profile types 2–4 vary in shape and size according to the degree of swelling of the herniated matrix, covered by the IMM, as well as by the associated change in the width of the tear in the OMM. These features are commonly present in the images of the type 2 profiles (Figs. 2 and 3). Thus, it is possible to capture a spectrum of configurations of type 2 profiles by using the transmission electron microscope (TEM). Type 3 profiles exhibit fragments of mitochondrial crista(e), while type 4 profiles do not contain cristae (profiles E and F in Fig. 1, and profiles 3 and 4 in Fig. 3). Type 4 profiles can be recognized by comparing the aspect of the swollen matrix with that (those) observed in the closely positioned types 2 and 3 profiles. However, since this type of profile often cannot be definitively distinguished from other unimembranous structures in the cell, it would not be possible to undertake morphometric analyses of type 4 profiles.

Mitochondria with ruptured outer membranes were found in drug-stimulated cells with typical apoptotic nuclear phenotypes, and also in companion cells with unaltered nuclear structures. Since, the release of MPIS into the cytoplasm has been considered as the point of no return of the programmed cell death process (Green and Kroemer, 2004; Kroemer et al., 2007) and because rupture of the OMM releases MPIS into the cytoplasm, we considered those companion cells with unaltered nuclear structure that contained mitochondria with ruptured outer membrane to be preapoptotic cells, as has previously been indicated in a similar context (Angermüller et al., 1998); their designation as preapoptotic will therefore be applied from here on.

**Fig. 4:**
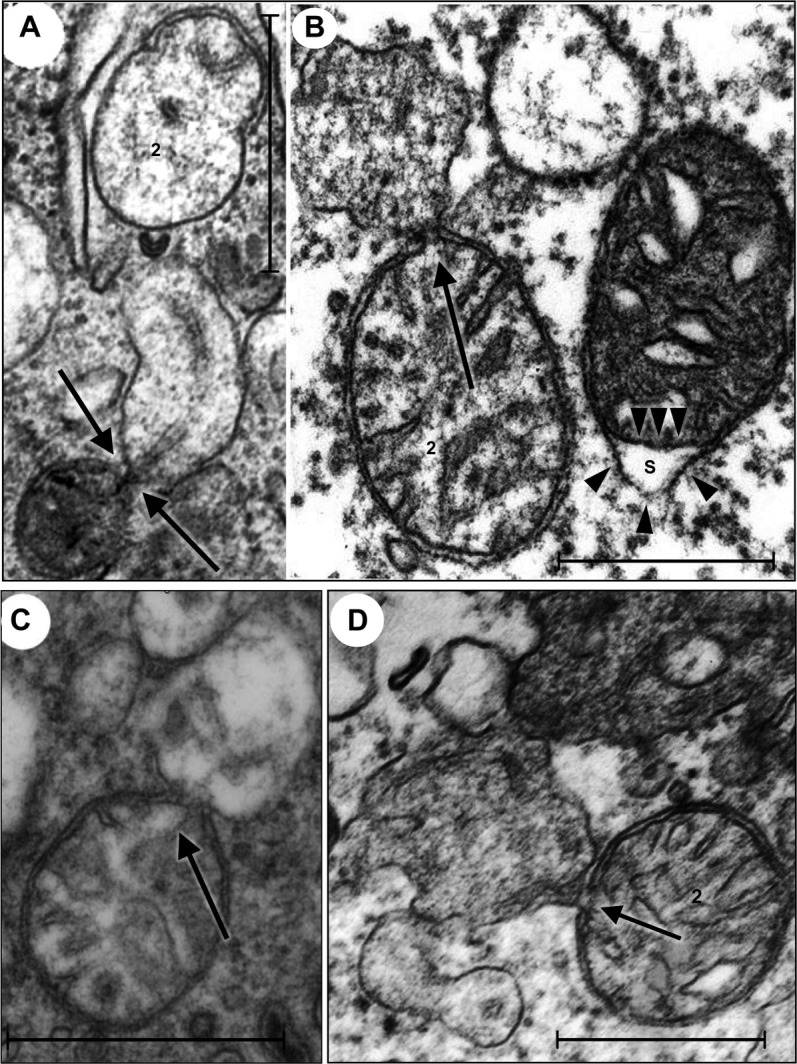
Minute sized, from 6 to 55 nm, ruptures of the OMM. The very dense region of the punctual rupture in panel **A** (opposing arrows) measures ∼6 nm or less if one considers its lowest part close to the mitochondrial matrix. Arrows in panels **B**–**D** indicate ruptured regions of the OMM with lateral dimensions of ∼30, 55, and 45 nm, respectively. In panel **B** (arrow), the channel width varies between 20 and 30 nm. In panel **C**, the perforation in the OMM (arrow) is partly obliterated by portions of the cristae. In panel B the mitochondrial profile at right has a nonswollen matrix and a localized intermembranous swelling marked (s). Possibly this profile represents the initial stage of a sequence which proceeds in Figs. 7 and 8. Panels A–C are from apoptotic PC 12 cells deprived of sera for 16, 8, and 4 hr, respectively. **D**: BHK 21 cell exposed to 6. μM camptothecin for 16 hr. Note: Panel C and D are from images published in the Braz J Morphol Sci (2006), 23, 57–74. These images are reproduced here with the appropriate permission of the Editor of the BJMS, which is no longer directly accessible online from the journal.

We observed rupture of the OMM with extensions of ∼6–55 nm (Fig. 4, arrows in panels A–D). The perforation (∼6 nm) of the OMM (opposing arrows in panel A, Fig. 4), represented by drawing B in Fig. 9, could correspond to the open form of the MPT pore, or to a widening of the original pore. In panel B of Fig. 4, the right mitochondrial profile exhibits an intermembrane swelling with a triangular profile. The IMM is at the base of the triangle (marked “s”), covering a mitochondrial matrix showing no signs of swelling.

**Fig. 5:**
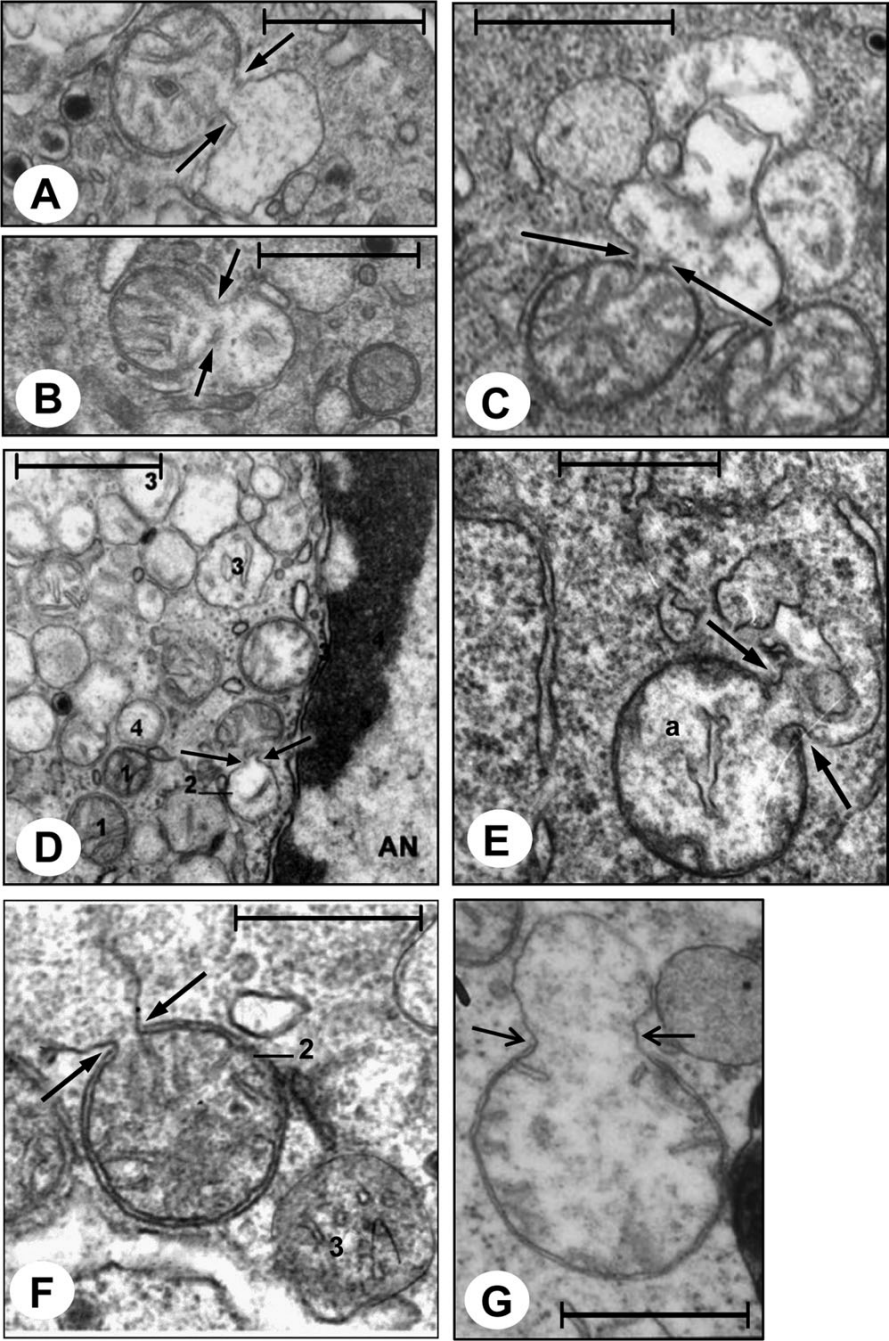
Small ruptures of the OMM with widths 30–100 nm in panels **A**–**F** (opposing arrows). Panels A–D exhibit type 2 profiles from 4 hr sera deprived PC-12 cells. The profiles in A and C pertain to the same microscopic field and the profile in B was found in a nearby thin section of the same cell. E and G, mitochondria from a preapoptotic and an apoptotic (**G**) Hela cell exposed to 10 ng/mL TNFα for 23 hr. F- Apoptotic PC-12 cell sera deprived for 16 hr.

In panels A–G of Fig. 5, the widths of the OMM perforations measure from ∼30 nm (panel C) to ∼100 nm (panel F). In mitochondria with small ruptures of the outer membranes, the degree of swelling of the matrix in regions with both mitochondrial membranes varied. In some cases, despite the amount of cristae being scant and the swelling spread throughout, no major stretching of the OMM could be observed (“a” in panel E, Fig. 5). Panel G shows a relatively common width of rupture of the OMM.

**Fig. 6:**
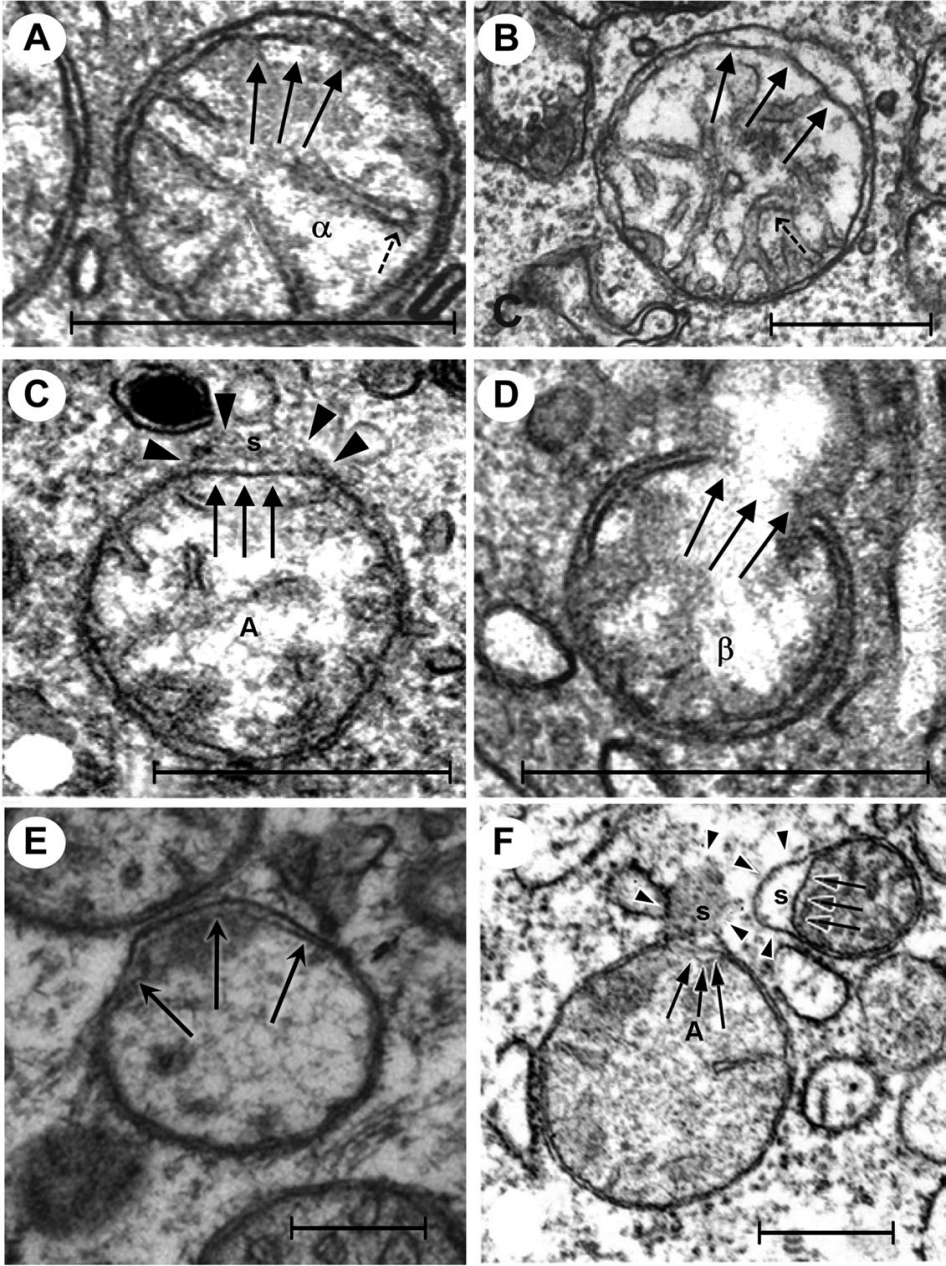
Incipient and more voluminous localized mitochondrial intermembrane swelling (triple arrows point to the IMMs). Incipient intermembrane swelling with thin, dense, parallel striae-like elements bridging the intermembrane space (**A** and **E**). Moderately engorged intermembrane swelling (**B**). Markedly swollen intermembrane space delimiting a triangular profile marked (s) (**C**). Rupture of the OMM with a width compatible of this profile having originated from a profile like in C (**D**). In the center, dome-like intermembrane swelling delimited by three small arrows pointing to the IMM and by three arrowheads demarcating a sector of the OMM; at the upper right an intermembrane swelling (s) situated over a mitochondrial matrix incipiently swollen (**F**). A, C, and D are from apoptotic 4 hr sera deprived PC-12 cells. B is from a preapoptotic HL 60 cell treated with 2 μM camptothecin 2 μM plus TNF alpha 100 ng/mL for 16 hr. E and F are from preapoptotic and apoptotic serum deprived BHK-21 cells exposed to 6 μM camptothecin for 16 hr.

**Fig. 7:**
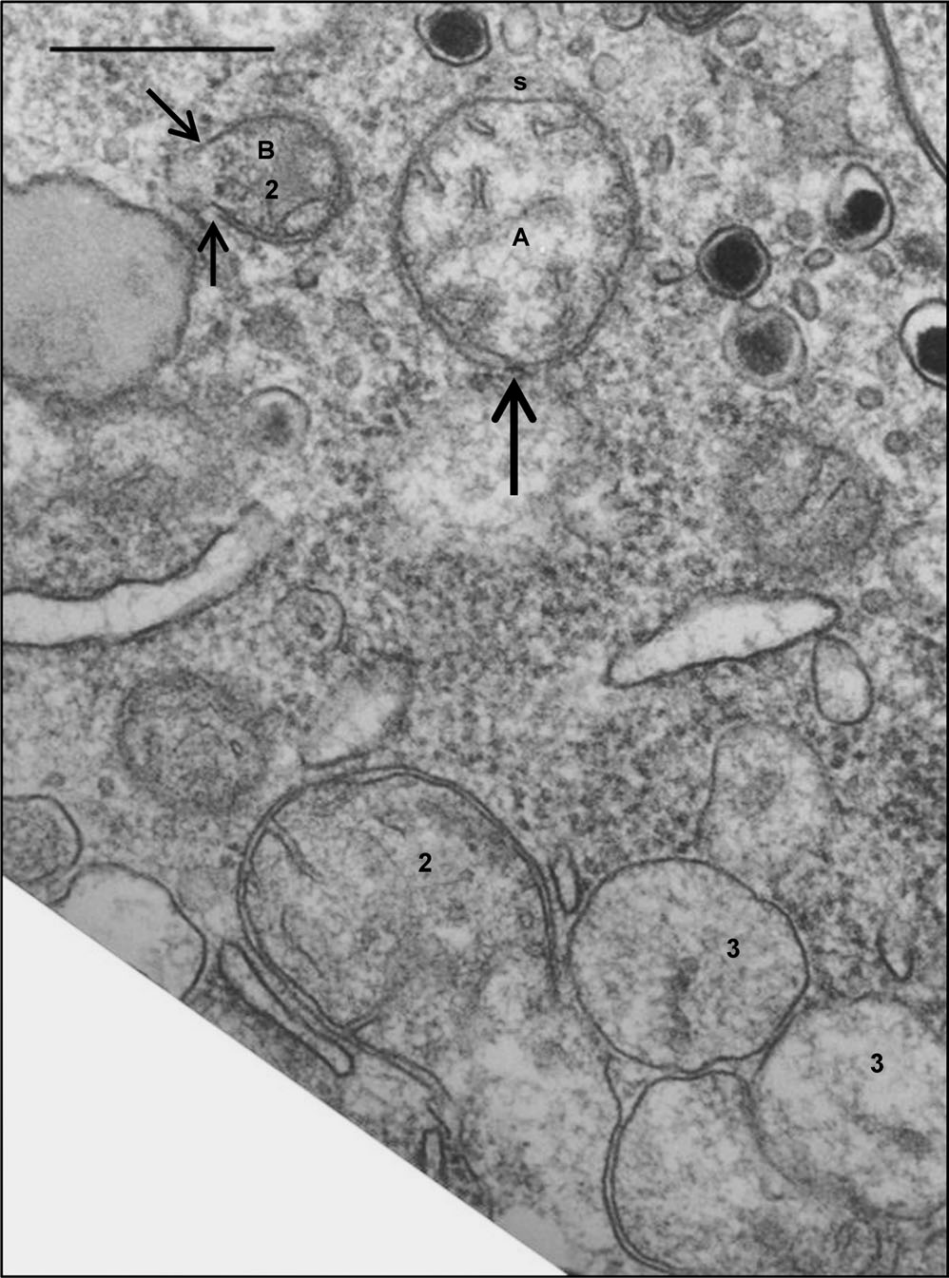
A wider view of panel C in Fig. 6. Profile B (arrows) may have the configuration that profile A (vertical arrow) would assume once the OMM ruptures.

**Fig. 8:**
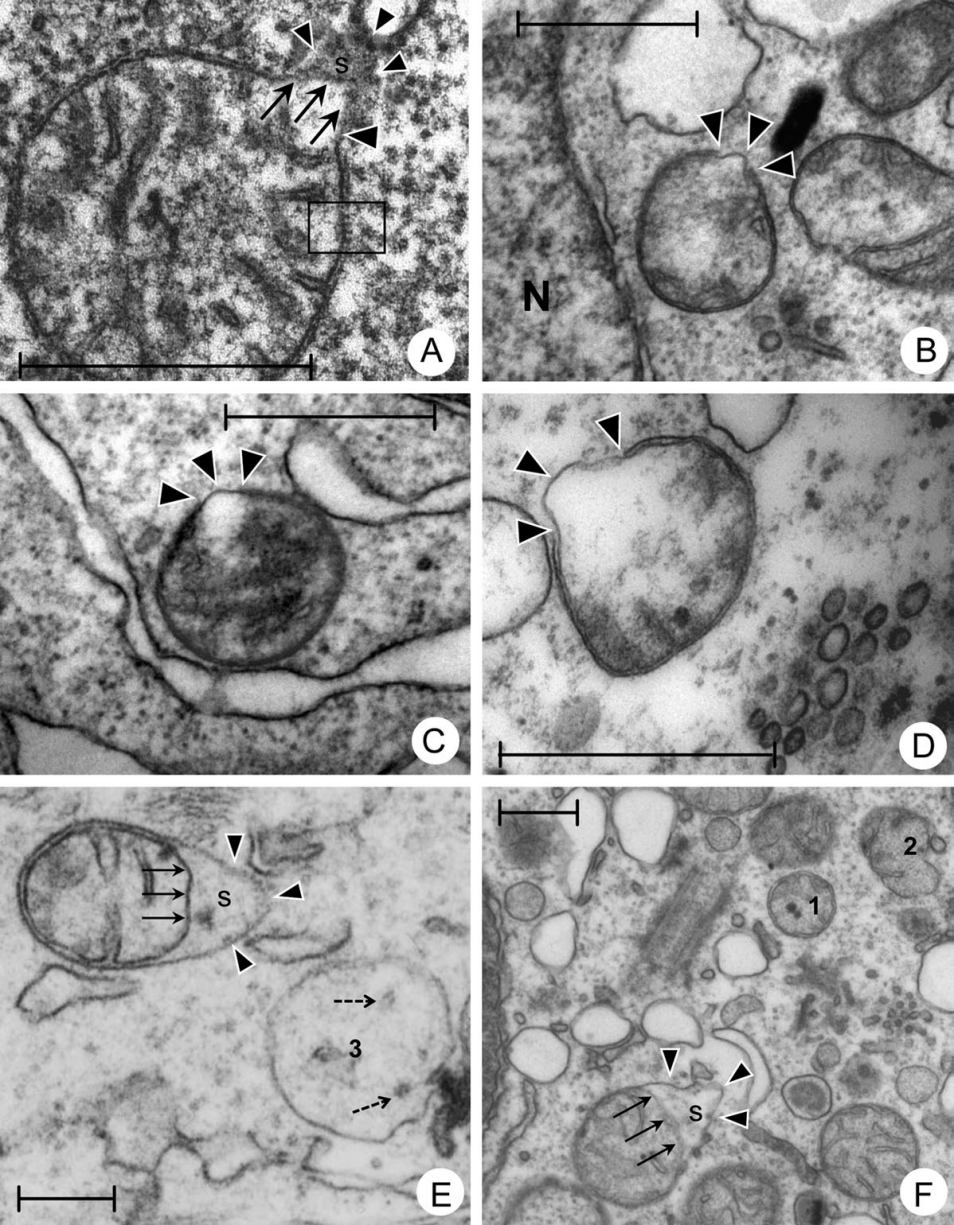
Arrows (IMM) and arrow heads (OMM) delimit the dome- and triangular-like sectors of profiles in **A**, **E**, and **F**, respectively. In E and F the triangular-like profiles are close to types 3 and 2 mitochondrial profiles and exhibit swollen mitochondrial matrices. In A it is unclear whether in the small square at right the parallel striae could represent channels crossing the intermembrane space. **B**–**D** Arrow heads point to small herniations of the mitochondrial matrix lined by the MMM. A BHK-21 cell with camptothecin 6 μM/16 hr; B–D HL-60 cells treated with TNFα 100 ng/mL plus camptothecin 2 μM for 6 hr; (E) PC-12* cells exposed to brefeldine A 2 μM plus staurosporine 0.5 μM for 16 hr. (F) HL 60 cell exposed to actinomycin D 0.25 μg/mL plus cycloheximide 6.25 μg/mL during 16 hr.

In some mitochondria (Figs. 6–8), a small segment of the space between the inner and outer membranes appeared to be swollen. The degree of swelling in these localized regions varied from incipient (panels A and E in Fig. 6) to massive (regions indicated by “s” in panel B in Fig. 4; panels C in Fig. 6; vertical arrow in Fig. 7; and panels E and F in Fig. 8). Some of these markedly swollen intermembrane sectors displayed OMM inflections that confer a triangular- [“s” in panel B in Fig. 4, panel C of Fig. 6, upper-right profile (vertical arrow) in Fig. 7, and panels E and F in Fig. 8] or dome-like (“s” in the large, central profile of panel F in Fig. 6, and in panel A of Fig. 8) profile to these sections. The width of the IMM separating the regions with dome- or triangular-like profiles, as measured from the mitochondrial matrix, amounted to ∼110 and 250 nm, respectively. The inner membrane boundary of the regions with a triangular-like profile appeared flattened against the adjacent mitochondrial matrix (3 arrows in the matrix pointing to the IMM and 3 arrow heads indicating the OMM of the profile sectors marked “s” In Figs. 4, 6, and 8). The small protrusions of the IMM (arrowheads, Fig. 8B–D) through the perforation in the OMM suggest that the initial rupture and the preceding intermembrane swelling were both localized and small.

The intermembrane space associated with the smallest intermembrane swellings detected, in panels A and E of Fig. 6, exhibit dense parallel striae-like structures that remotely resemble the gap junctions seen in thin sections. No other images similar to these could be obtained. While the mitochondrial matrix adjacent to the intermembrane swelling marked “s” in panel B of Fig. 4, exhibits no sign of swelling, a somewhat similar dense matrical region shows incipient swelling (upper right profile in panel F of Fig. 6). The altered mitochondrial profiles in Figs. 6–8 suggest that the initial ruptures of the OMM may also occur in the width range, ∼100–250 nm.

**Fig. 9:**
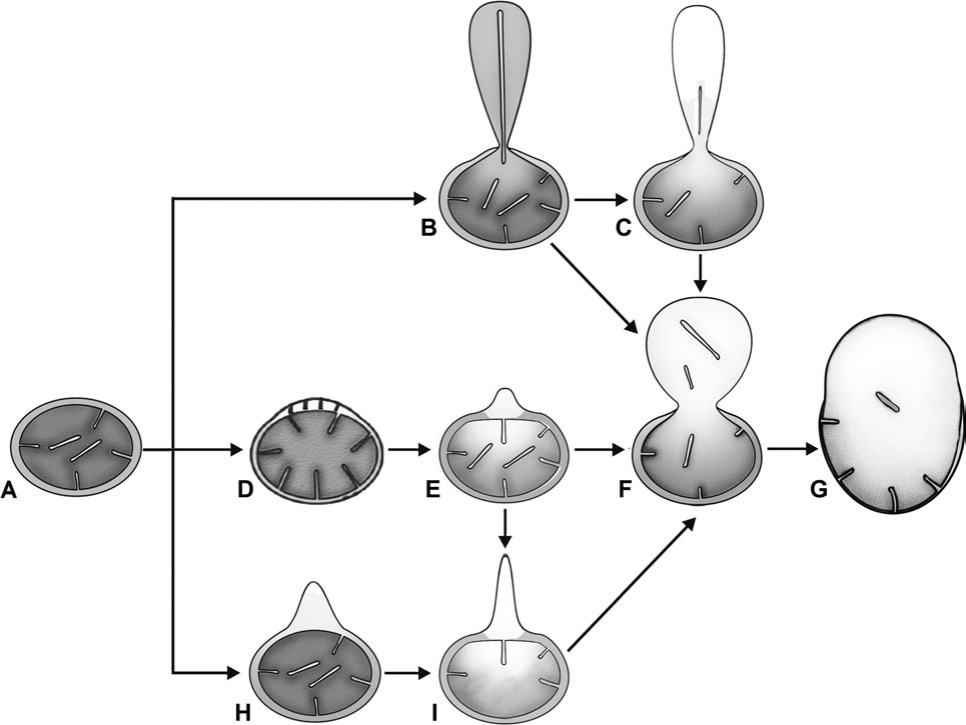
Stages possibly preceding and following the rupture of the OMM are tentatively schematized. The depicted profiles are based on actual electron micrographs. Stages **A–C** and/or **A**, **B**, **F** represent initial ruptures of the OMM within the widths of ∼6–100 nm. Stages **A**, **D–G** or **A, H, I, F** and **G**, represent likely sequences leading to ruptures of the OMM in the range of some 100–250 nm. **B** opposite arrows, panel A Fig. 4; **C** panels B–D in Fig. 4, **D** panels **A** and **E** in Fig 6, **F**, **E** and **I** (marked s in panel C in Fig. 6 and panels E and F in Fig. 8), panel **F** (upper profile 2 in Fig. 3 and inset in Fig. 2), **H** panel B Fig. 4 [profile marked as (s)]; **G** lower and upper profiles 2 in Fig. 3 and upper right profile in panel A in Fig. 4, respectively.

The structural alterations that are suggested to follow and eventually to precede the moment of rupture of the OMM shown in Figs. 3^8^ are reproduced schematically in Fig. 9. Drawings B–G are derived from actual electron micrographs. Some of the transitions in mitochondrial forms were observed (B, C, F, and G), while others are likely possibilities (D–G; H, I, F, and G; H, I, F, and G).

## DISCUSSION

### The Initial Rupture of the OMM Appears to Occur Within Two Ranges of Widths

Just before the rupture of the OMM, the permeability of both mitochondrial membranes apparently increases where the localized intermembrane swelling is forming (in the range, 100–250 nm). It is undetermined whether the Bax and Bak pro-death proteins localized in the OMM surface (Vogler et al., 2008) have a role in this permeabilization. Here, we observed without exception that the accumulation of intermembrane fluid appeared to have flattened the IMM bordering the swelled mitochondrial matrix (Figs. 6 and 8). It appears that although the selective permeability of the IMM was maintained in most cases, it allowed the entrance of sufficient fluid into the matrix to swell it (Figs. 6^8^). The intermembrane swelling shown in the conical or triangular profile marked “s” in panel B of Fig. 4 is intriguing. The matrix subjacent to the flattened IMM, which is the base of the triangle, showed no signs of swelling. It is unclear whether this particular triangular profile is revealing that only some of the participants that act in the mechanism that leads to apoptotic rupture of the OMM (those with widths of 100–250 nm) are present. Further, this profile might represent the initial stage of a sequence in which the mitochondrial matrix subjacent to the intermembrane swelling is starting to swell (“s” in Fig. 6F). The swollen mitochondrial matrices bordering the regions indicated by “s” in panels E and F of Fig. 8 might represent a progression of the previous stage. It is unclear whether the pronounced increase in curvature of the OMM occurring in these regions is one causative agent of the rupture of this membrane. Similar intermembrane triangular regions were described in Drosophila cells expressing the proteins Reaper and Hid, which are IAP-binding motif-containing positive regulators of developmental apoptosis (Abdelwahid et al., 2007). The mitochondria from these cells were described as disrupted. Consistent with theoretical predictions, the insertion of Bax-type proteins into a planar lipid increases the monolayer curvature, forming a large protein-lipid pore (Basañez et al., 1999, 2002). The additive actions of proteins from the OMM, along with Bid and Bax, induced the formation of 25–100 nm pores in liposomes (Schafer et al., 2009).

The punctate rupture of the OMM (opposing arrows in panel A Fig. 4), which exposes an IMM in permeability transition to the cytoplasm, is sufficient to produce the formation of a focal hernia of considerable size. Here, the herniation was the longest observed and was cylindrical. We could not determine whether small ruptures of the OMM of ∼30–55 to 100 nm arose from the expansion of the ∼6 nm-wide hole or from localized small intermembrane swelling. We also do not know to what extent the spectrum of small apertures observed in the OMM relate to the following processes as: (a) possible crosstalk between the mitochondrial apoptosis-induced channel and the aperture of the MPT pore (Kinnally and Antonsson, 2007, Kinnally et al., 2010); (b) the formation of the MPT pore in a disrupted lipidic sector of the cell membrane in which disassembly of the lipid phase would be promoted by the oxidation caused by higher levels of reactive oxygen species (ROS) (Kowaltowski et al., 2001); and (c) the formation of a lipid-protein pore associated with an increase in the curvature of the OMM (Basañez et al., 1999, 2002) (note marked inflections of the OMM in panel C Fig. 6 and panels E and Fig. 8). Of note, in the lower part of panel A in Fig. 4, the curvatures of the inner and outer membranes, the centers of which contain the punctual rupture (opposing arrows), are greater than elsewhere in the profile.

### The Opening of the MPT Pore and Subsequent Rupture of the OMM Are Complex Events

The irreversible opening of the MPT pore and the subsequent rupture of the OMM observed *in vitro* (Petit et al., 1998) are considered as nonspecific events (Armstrong, 2006). This concept stemmed from the idea that the rupture of the OMM is caused by excessive stretching of this membrane promoted by an expanded IMM and the maximally swollen mitochondrial matrix. This mechanism of rupture following MPT is considered a universal phenomenon (Gogvadze et al., 2006; Rasola and Bernardi, 2007). This concept is supported by findings that focal hernias similar to the ones presented occur in isolated mitochondria exposed to hypo-osmotic media (Hackenbrock, 1966; Stoner and Sirak, 1969).

The observations that the rupture of the OMM is often very small and unrelated to a previous associated large matrical swelling raises pertinent questions. What are the chemical and physical participants in the mechanism that drills small holes in the OMM? Would these participants be the same if the initial perforations of the OMM occur in two width ranges, ∼6–55 or 100 nm and 100–250 nm? The loss in the selective permeability of the IMM is a necessary participant in generating small ruptures in the OMM. Thus, these ruptures derive from factors that determine the process of the opening of the MPT pore and from processes that modify or block them. An increasing number of positive and negative regulators of cell death have been identified that control the opening of the MPT pore (Grimm and Brdiczka, 2007; Rasola and Bernadi, 2007). Among the factors involved in the modulation and/or permanent aperture of the MPT pore and rupture of the OMM, three groups are of key importance. The first group is represented by the signaling protein kinases that mediate the command for the cell to commit suicide via the mitochondria (Armstrong et al., 2006; Gogvadze et al., 2006, Grimm and Brdiczka, 2007; Rasola and Bernardi, 2007; Indran et al., 2011). Downstream of the network of kinases are situated the effectors of events represented by the increase of intra-mitochondrial levels of Ca^2+^ and of ROS (Ott et al., 2007; Lemasters et al., 2009) and activation of the pro-apoptotic proteins of the Bcl-2 family, such as Bax and Bak (Tafani et al., 2001; Sharpe et al., 2004), Bid (Li et al., 1998; Garcia-Perez et al., 2012) as well as p53 (Liu et al., 2008; Charlot et al., 2004). The activation of these proteins correlates with the aperture of the MPT pore under various experimental conditions. For example, the activation of p53 results in the up-regulation of Bax and its translocation to the surface of the OMM where it directly or indirectly induces the aperture of the MPT pore (Karpinich et al., 2002) and promotes cell death.

### The Loss of the Selective Permeability of the IMM in Apoptotic Cells

A relevant question is whether the *in situ* loss of selective permeability of the IMM is as rapid as the shutdown of an electric current, or whether it is initially lost only in the region where rupture of the OMM occurs. In this context, it is also possible that the transmembrane potential (ΔΨm) is temporarily maintained in the remaining mitochondrial body in which both membranes are still intact, even while the IMM is being extruded into the cytoplasm (profiles B, C, and F in Fig. 9).

It appears that the time required for spreading of the region of the initial loss in permeability along the entire IMM is dissimilar among mitochondria. Observations of the variations in ΔΨm in an individual mitochondrion on a time-resolved scale indicate that in a portion of the mitochondria, the MPT pore opening induces a rapid depolarization, while the remaining portion undergoes a slower, progressive depolarization (Higuchi et al., 2005).

### In the Mitochondrial Outer Membrane Permeabilization or Mitochondrial Membrane Permeabilization Models, the Release of the MPIS to the Cytoplasm Necessarily Precedes the Formation of the Opening of the MPT Pore

When exposed to the cytoplasm by rupture of the OMM, an IMM devoid of its selective permeability is the origin of a focal hernia. The rupture of ∼6 nm in the OMM (panel A in Fig. 4) is the only rupture with such small dimension obtained after examination of several thousands of mitochondrial profiles in apoptotic and in preapoptotic cells. From this smallest channel originated the lengthiest, swollen type of herniation that was observed. An equivalent focal hernia would be formed on the mitochondrial surface if the cytoplasm were directly exposed to a permeable IMM through channels. Thus, under the premise of eviction of proteins of the intermembrane space to the cytoplasm through a nonruptured OMM the formation of the MPT pore cannot be the initial event of activation of the mitochondrial apoptotic pathway; and indeed, it is not. The late appearance of MPT guarantees that the IMM remains impermeable to various important ions and may be exposed to the cytoplasm through newly formed or opened channels, without consequences.

Identification of the loss of the selective permeability of the IMM can be faultlessly done at the single mitochondrial level by the recognition that the outer membrane is ruptured. The permeabilization of the IMM also promotes the disappearance of the mitochondrial fluorescence due to cationic probes. In apoptotic cells exposed to 3 μM STS, it was noted that after an early release of MPIS, the ΔΨm is maintained not from the normal activity of the electron transport chain but from a retrograde electron flow (Düssmann et al., 2003).

The MOMP model is mentioned or implied in numerous publications as depicting the prevalent manner by which MPIS leave the mitochondria to activate the mitochondrial apoptotic pathway. However, in a significant proportion of cases where apoptosis is induced by various agents, the release of MPIS is promoted by the opening of the MPT pore. It is therefore necessary to re-evaluate the concept that MMP or MOMP is the main means by which MPIS are released into the cytoplasm of apoptotic cells. Clearly, searching for mitochondria with ruptured outer membrane by TEM as part of future studies, in which the kinetics of the release of MIPs and the appearance of MPT are evaluated sequentially at very short time intervals, can make a valuable contribution to elucidating why MPT precedes the release of MPIS into the cytoplasm in so many apoptotic cells, while in other experimental settings, the opening of the MPT pore follows the discharge of MPIS from the mitochondria.

## Abbreviations used

IMM: inner mitochondrial membrane
MOMP: mitochondrial outer membrane permeabilization
MPIS: mitochondrial proteins of the intermembrane space
MPT: mitochondrial permeability transition
OMM: outer mitochondrial membrane
ROS: reactive oxygen species
TEM: transmission electron microscope
